# A self-assembled photoresponsive gel consisting of chiral nanofibers

**DOI:** 10.3762/bjoc.14.174

**Published:** 2018-08-01

**Authors:** Lei Zou, Dan Han, Zhiyi Yuan, Dongdong Chang, Xiang Ma

**Affiliations:** 1 Key Laboratory for Advanced Materials and Institute of Fine Chemicals, School of Chemistry & Molecular Engineering, East China University of Science & Technology, Shanghai 200237, China

**Keywords:** chirality, nanostructure, organogel, photoresponse, self-assembly

## Abstract

A novel compound based on a glutamic acid skeleton, containing azobenzene as a photoresponsive group and ureidopyrimidinone (UPy) as a connection site, was designed and synthesized. The monomer is capable of forming an organogel in nonpolar organic solvents and different types of nanostructures in other solvents. The state of the gel and the chirality of the nanostructures could both be adjusted by subsequent light irradiation at different wavelengths. The helical nanofiber-like morphology was verified in the internal structure of the gel. The performance of this gel was investigated by a series of methods, such as UV–vis absorption spectroscopy, circular dichroism, scanning electron microscopy and rheological techniques. This work provides a new method for facile synthesis of chiro-optical gels.

## Introduction

Supramolecular gels [1–2] immobilized by three-dimensional networks through self-assembly have drawn significant attention in the past decades. They are normally fabricated by means of noncovalent intermolecular interactions [3], such as π–π stacking, hydrogen bonding, van der Waals forces, hydrophobic, electrostatic, host–guest and other interactions. Interestingly, some of them can be assembled into distinctive nanostructures through gel formation [4–6].

Various functional nanostructures have shown great potential for applications in many important areas, for example, nanofabrication [5,7–10], drug delivery [11–12], and chemosensing [13–14]. Among the supramolecular gels, the low-molecular-weight gels (LMWGs) [15–16] are those that self-assemble into gels in organic solvents with molecular weights of <2000 Da. The weak noncovalent intermolecular interactions between LMWGs make them more sensitive to external conditions [6,17–19], such as solvent, light and temperature. These characteristics meet currents demands for conveniently controlling the assembly of materials according to their size, shape, and morphology.

Chiral functional materials have aroused much attention for their potential applications. Liu and co-workers [20–31] have built a multifunctional controllable gel system, which utilized L-glutamic lipid to construct nanofibers, nanotwists, and nanotubes with the property of chirality. Azobenzene, which is structurally photosensitive, is widely chosen to construct optically controlled systems [17,30,32–35]. This moiety is also frequently employed as a building block because of its strong π–π stacking in nonpolar solvents.

**Scheme 1:**
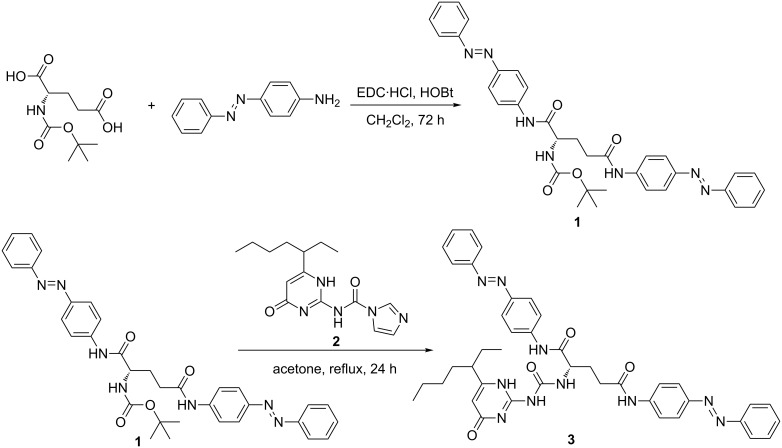
The preparation of compound **3**.

**Figure 1:**
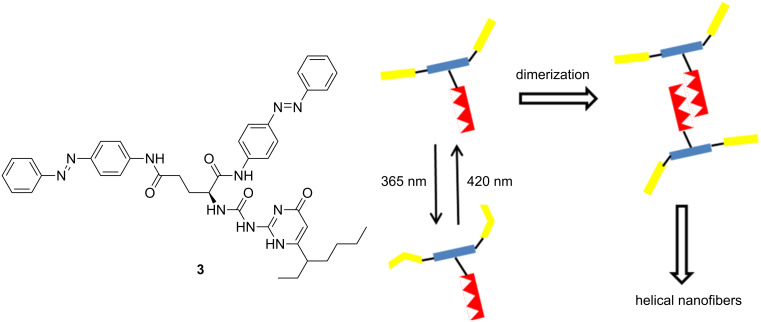
The chemical structure and the schematic representation of compound **3** as well as the proposed assembly process of the nanostructure.

Herein, a novel compound **3** containing both chiral L-glutamic lipid and azobenzene was designed and synthesized (Scheme 1). It is used as a candidate to form a new chiro-optical system [30,36–41]. Ureidopyrimidinone (UPy), as a connection site, is also introduced to make quadruple hydrogen bonding [42–44]. The structure and schematic representation of **3** are shown in Figure 1. The possible assembly process of the nanostructure is proposed as well. It is found that compound **3** is able to form a gel in nonpolar solvents. The assembled structures of **3** in different solvents were also investigated. The photoresponsiveness of the formed nanostructure was investigated concomitantly as well.

## Results and Discussion

The monomolecular compound **3** can be easily synthesized in 3 steps. The compound has the necessary features for gel formation. The quadruple hydrogen bonding created by UPy moieties is quite stable in nonpolar solvents. Therefore, the molecule can easily assemble into dimers. Then, the *trans*-isomers of azobenzene can stack with each other via π–π interactions. Further, the acylamino group of the glutamic acid moiety at the center of the molecule also promotes this aggregation through hydrogen bonding interactions. Finally, the chirality of glutamic acid may be magnified along with the formation of supramolecular structure.

**Figure 2:**
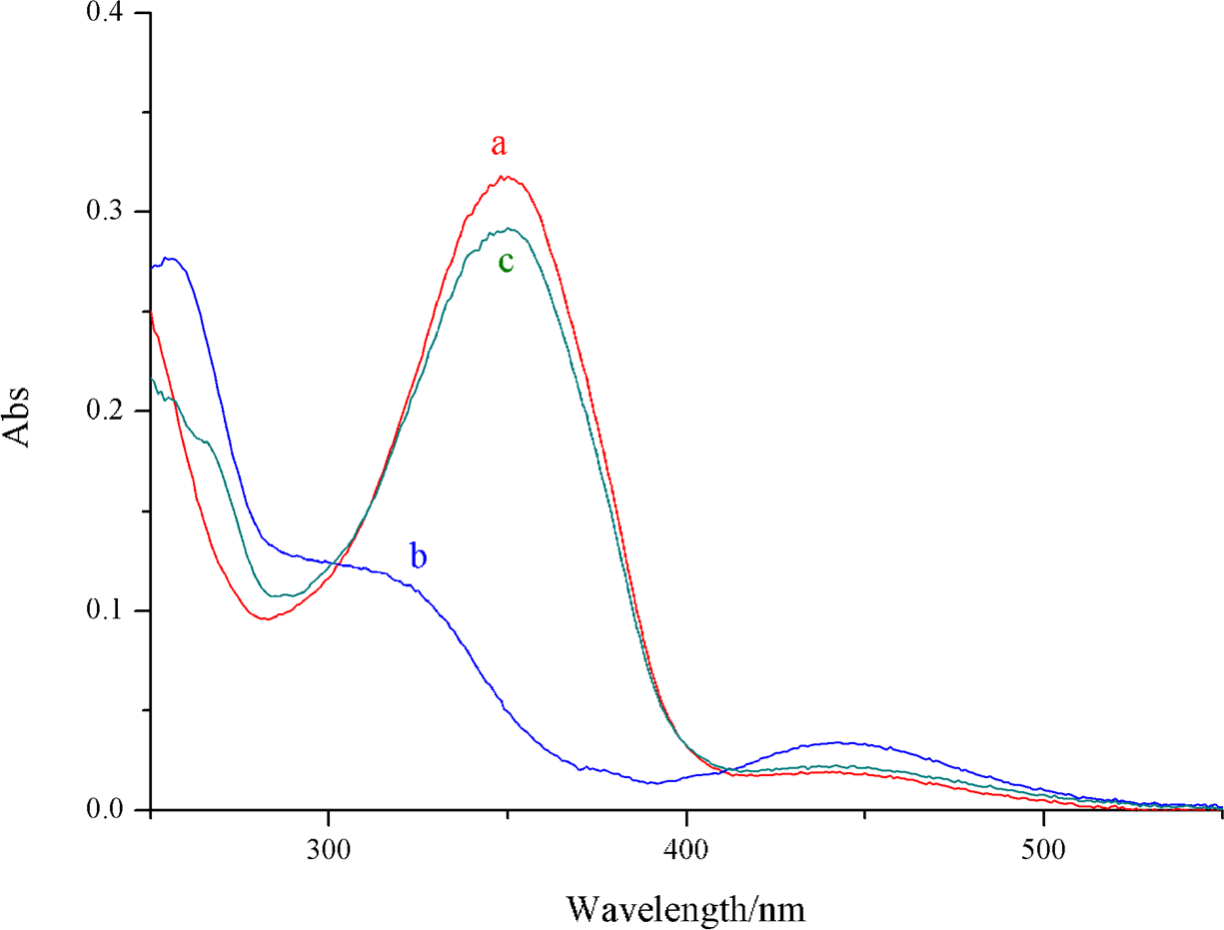
UV–vis absorbance spectra of a) compound **3** and b) irradiated by a light source of 365 nm and c) then treated by light of 420 nm. The concentration was 1.0 × 10^−5^ M in CHCl_3_.

To investigate the potential photoresponsiveness of compound **3**, the UV–vis absorption spectrum was measured to trace photochemical and photophysical properties of the solution of compound **3** (1.0 × 10^−5^ M in chloroform). As shown in Figure 2, the azobenzene *trans*-isomer displayed a strong absorbance peak at 352 nm. When exposed to ultraviolet light of 365 nm, the peak at 352 nm obviously decreased and reached a photostationary state within 5 minutes. The equilibrium could be reversed by subsequent exposure to visible light (420 nm) and UV light (365 nm) irradiation, whereby the equilibrium could be reached within 6 min and 4 min, respectively. However, the fatigue durability of this compound did not meet the expected requirement; after two cycles of light irradiation, the photoresponsiveness was clearly weakened. This defect may be ascribed to the rigid structure of compound **3**. These two azobenzene moieties within the molecule are close and the dimerization of the UPy moiety results in a more crowded structure.

**Figure 3:**
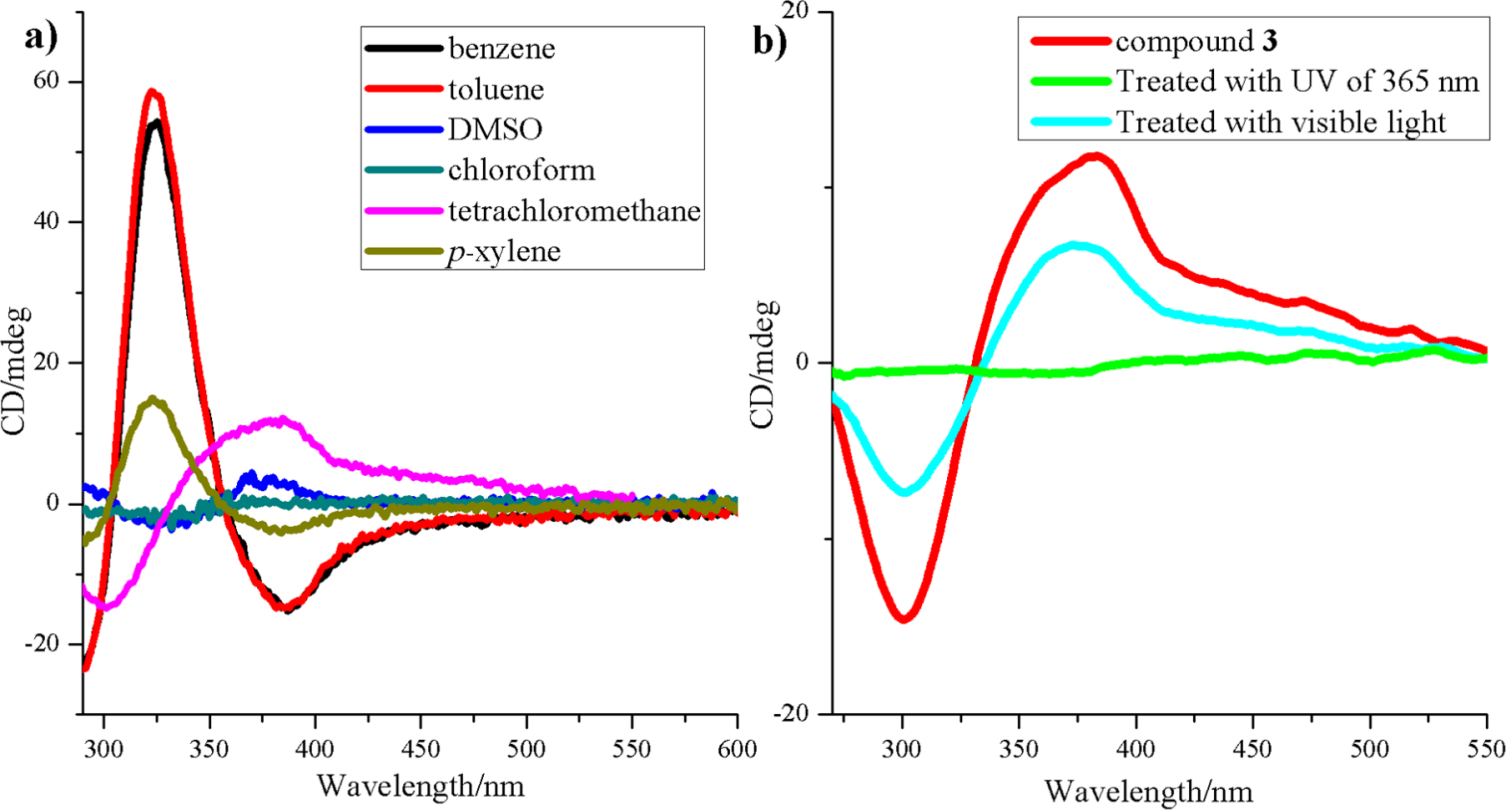
CD spectrum of compound **3** in solutions of a) benzene, toluene, *p*-xylene, chloroform, tetrachloromethane and DMSO with identical concentration (3.0 × 10^−5^ M), b) before and after being exposed to UV (λ = 365 nm) and visible (λ = 420 nm) light.

The circular dichroism (CD) spectrum was then measured to trace the different states of aggregation in solution of compound **3** in different solvents. Benzene, toluene, *p*-xylene, chloroform, tetrachloromethane and DMSO were chosen as solvents to prepare solutions with uniform concentration (3.0 × 10^−5^ M). As evidenced in the CD spectrum (Figure 3), the CD signal in a solution of DMSO and chloroform was very weak, and the CD signal in tetrachloromethane and *p*-xylene was not very strong but discernible. On the other hand, the CD signal in aromatic solvents, like benzene and toluene, was strong enough to confirm the formation of chiral structures. These results indicate that the chiral nanostructures can be only obtained in solvents with lower polarity, especially in aromatic solvents.

To study the chirality of compound **3** and its reaction to light stimulation, **3** (in CCl_4_) was exposed to visible irradiation or UV light. The CD spectrum was then recorded. In Figure 3b, two obvious peaks at 300 nm and 375 nm evidence the existence of the chiral structure in the solution. This CD signal disappeared after irradiation with light at 365 nm, implying that the chiral structure in the solution had been destroyed. The obvious chiral signal reappeared when the same solution was exposed to visible light of 420 nm before reaching a stable state. However, this process was partially reversible. The compound **3** cycling test, with more than 8 cycles, caused the total disappearance of the CD signal and the process was then no longer reversible (Figure S8, Supporting Information File 1).

**Figure 4:**
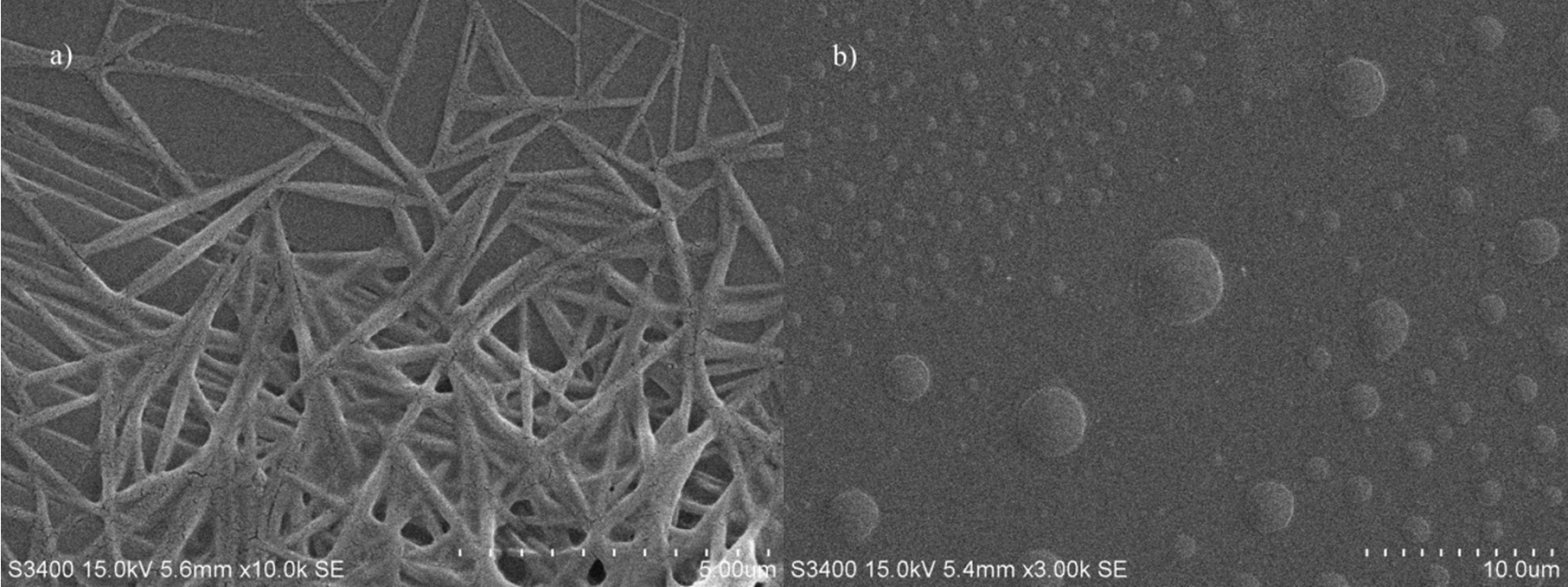
SEM images of the microstructure a) obtained by the self-assembled compound **3** in CHCl_3_ on the surface of mica and then b) treated with UV light (365 nm).

The morphology of compound **3** was further characterized by scanning electron microscopy (SEM, Figure 4). The samples were prepared by evaporating the solution of compound **3** on a surface of mica, and the differences in various solvents were also investigated. The sample in DMSO did not show a chiral self-assembled nanostructure owing to the destruction of quadruple hydrogen bonding and π–π stacking in DMSO. However, beautiful acicular fibers could be detected on the surface treated with a chloroform solution, and a dendritic network was observed on the sample made from benzene solution (Figure S9, Supporting Information File 1). The photoresponsiveness of compound **3** was also investigated by drying the chloroform solution of compound **3**, which was exposed to sufficient UV light (365 nm) beforehand, on the surface of mica and detected by SEM, whereby only small discs were found as shown in Figure 4b. This implies that the UV light of 365 nm can efficiently destroy the self-assembled structure, and the nanostructures of the assembled compound **3** can be adjusted by both solvent and light.

**Figure 5:**
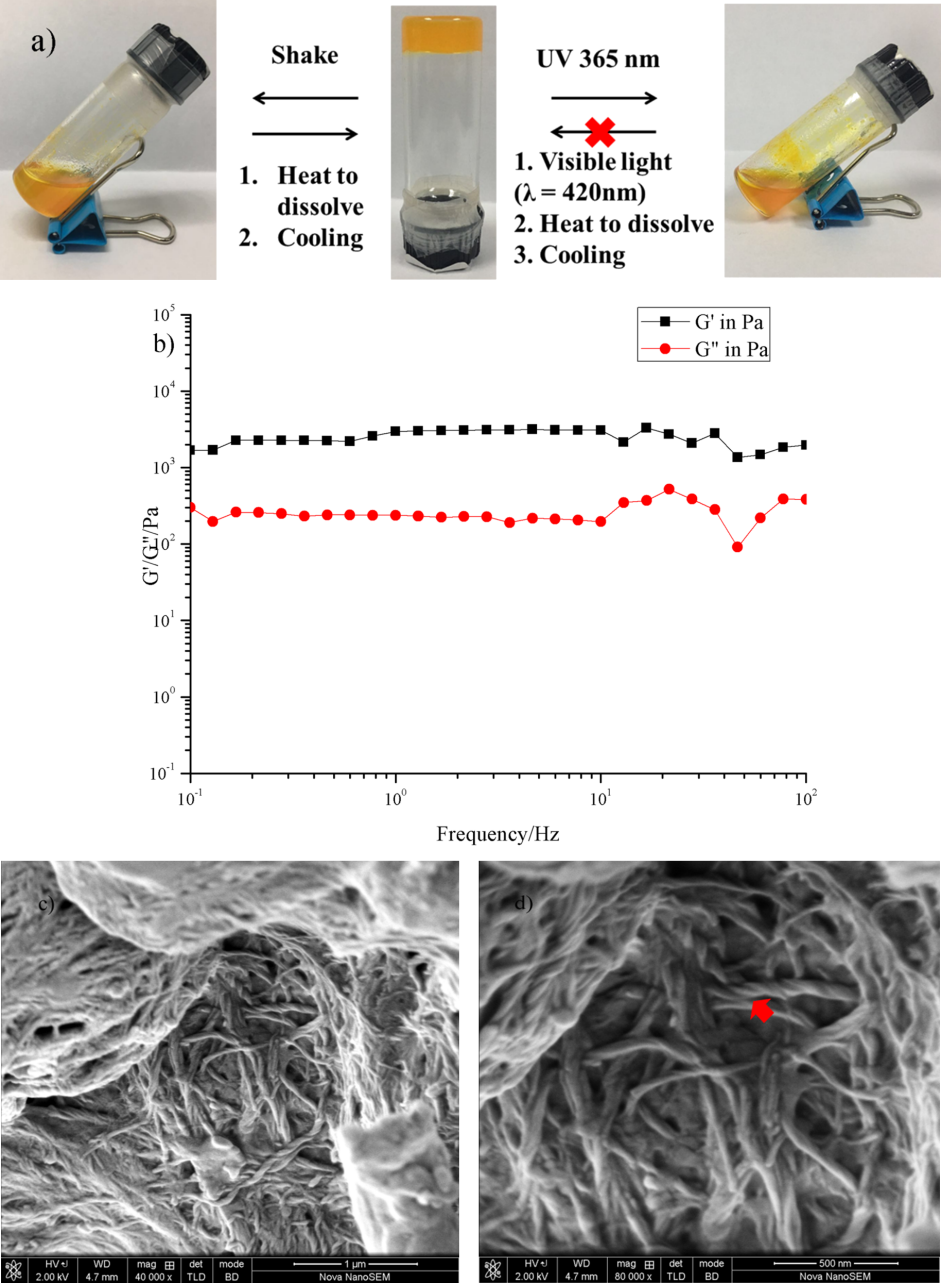
a) The gel-to-sol transformation of the samples via different routes. b) Dynamic frequency sweep of the gel fabricated in benzene. (c,d) FESEM images of compound **3** xerogels.

The formation of organogels in various solvents was conducted to test the gelation ability of compound **3**. The gel was fabricated by melting compound **3** in solution by heating for about 20 min, followed by allowing the solution to rest at room temperature for 30 min. The gelation ability of compound **3** in various organic solvents was investigated by using the “stable to inversion of a test tube” method (Figure 5a). Here we arrive at the conclusion that compound **3** can only form an organogel in nonpolar aromatic solvents such as benzene and *p*-xylene. Table 1 lists the solvent parameters and the gel-forming ability of compound **3** in various organic solvents. The critical gelation concentration (CGC) is also noted. We took the gel formed in benzene as the example for the following investigations. The rheological technique (Figure 5b) was used to measure the rheological properties of this gel system first. The dynamic frequency spectra of the gel indicated that the elastic modulus G’ was higher than the viscous modulus G” when the frequency ω was between 0.1 and 100 Hz. This showed that the system conforms to the character of the gel. However, the gel is not stable under shaking, where it is broken into a mixture of solution and small pieces. After melting these particles again then natural cooling, the gel state was once again obtained.

**Table 1:** Gelation ability of compound **3** in various organic solvents.

solvent	CGC^a^ [mg mL^−1^]

hexane	P
cyclohexane	P
CCl_4_	G (7.5)
benzene	G (6.5)
methylbenzene	HG
*p*-xylene	G (5.0)
CHCl_3_	S
CH_2_Cl_2_	S
THF	S
acetone	S
acetonitrile	S
DMF	S

^a^G = gel; P = precipitation; S = solution; HG = half gel.

Field emission scanning electron microscopy (FESEM) was utilized to characterize the xerogels obtained by freeze drying. As shown in Figure 5c,d, plenty of helical nanotwists could be found in the xerogel. These fibers were intertwined together to form a 3D nanostructure. This result partially showed the gel formation pathway. Compound **3** may form dimers at the beginning, which can be detected by high-resolution mass spectrometry (Figure S7, Supporting Information File 1). Then, the dimers and single molecules **3** were able to assemble together through π–π stacking and hydrogen bonding to fabricate the nanofibers. Finally, those fibers twisted with each other to form nanotwists of wider diameter.

Finally, the gel system was irradiated with ultraviolet light or visible light to test its stimuli response. Under 365 nm light exposure, the gel melted into a turbid liquid within 20 min. However, the system could not easily be reversed due to the poor reversibility of compound **3** in benzene that was investigated previously.

## Conclusion

We have synthesized a novel chiral compound containing azobenzene as the photoresponsive group and UPy as the connection site. The monomer was capable of forming chiral nanostructures and a low-molecular-weight organogel in nonpolar organic solvents. The monomer also can form different types of nanostructures in different solvents, and the kind of solvent was found to be crucial for the chirality of the assembled structure. The gel-to-sol process could also be modified by shaking and UV light. The rheological behavior of the gel was investigated and found to meet the basic requirement of a gel. The inner structure of the gel was determined to be a cross-linked network made of chiral nanotwists. This work provides a novel method to build chiro-optical soft material systems.

## Experimental

### Synthesis of compound **1**

1-Ethyl-3-(3-dimethylaminopropyl)carbodiimide hydrochloride (EDC·HCl, 8.04 g, 0.044 mol) and 1-hydroxybenzotriazole (HOBt, 5.94 g,0.044 mol) were added to a 200 mL CH_2_Cl_2_ solution of Boc-L-glutamic acid (5 g, 0.02 mol) and 4-aminoazobenzene (7.8838 g, 0.04 mol), then the obtained mixture was stirred at room temperature for 72 h under an Ar atmosphere. The obtained yellow solid was isolated by filtration and washed three times with CH_2_Cl_2_. The crude product was recrystallized from THF/H_2_O to yield compound **1** as a yellow solid (10.53 g, 87% yield). ^1^H NMR (400 MHz, DMSO-*d*_6_) δ 10.43 (s, 1H), 10.33 (s, 1H), 8.02–7.71 (m, 12H), 7.68–7.43 (m, 6H), 7.22 (d, *J* = 7.6 Hz, 1H), 4.18 (dd, *J* = 13.5, 7.9 Hz, 1H), 2.17–1.90 (m, 2H), 1.36 (d, *J* = 34.1 Hz, 9H); ^13^C NMR (100 MHz, DMSO-*d*_6_) δ 171.44, 170.83, 155.43, 151.99, 147.58, 147.35, 142.35, 142.05, 131.04, 130.97, 129.38, 123.64, 122.32, 122.28, 119.50, 119.17, 78.23, 54.78, 32.92, 28.17, 27.07, 25.09; HRMS *m*/*z*: [M + H]^+^ calcd for C_34_H_36_N_7_O_4_^+^, 606.2829; found, 606.2830.

### Synthesis of compound **3**

Compound **2** was easily prepared according to a literature method [45]. Five mL of trifluoroacetic acid was dropped into a 25 mL CH_2_Cl_2_ solution of compound **1** (1.0 g, 1.65 mmol), then the mixture was stirred at room temperature for 2 h under an Ar atmosphere. Evaporation of the resulting red solution was performed under reduced pressure, and small amounts of CH_2_Cl_2_ was frequently added to the bottle until the trifluoroacetic acid was removed entirely. The resulting yellow solid was added to an acetone solution of compound **2** (1.22 g, 4.03 mmol) under an Ar atmosphere, then the mixture was heated at reflux for 24 h. The evaporation (under reduced pressure) and further purification of the resulting solution was carried out by column chromatography using CH_2_Cl_2_/CH_3_OH (50:1, v/v) and CH_2_Cl_2_/CH_3_OH (10:1, v/v) to afford **3** as a yellow solid (620 mg, 62.5% yield). ^1^H NMR (400 MHz, DMSO) δ 11.29 (s, 1H), 10.67 (s, 1H), 10.34 (s, 1H), 9.68 (s, 1H), 8.30 (s, 1H), 8.01–7.68 (m, 12H), 7.68–7.43 (m, 6H), 5.77 (s, 1H), 4.64 (s, 1H), 2.22 (dd, *J* = 13.5, 6.0 Hz, 2H), 2.05 (dd, *J* = 13.6, 6.7 Hz, 1H), 1.55 (dd, *J* = 15.8, 10.0 Hz, 4H), 1.29–1.04 (m, 6H), 0.85–0.71 (m, 6H); ^13^C NMR (100 MHz, DMSO) δ 170.51, 151.99, 147.79, 147.34, 142.30, 141.65, 131.11, 130.96, 129.41, 129.37, 123.57, 122.34, 122.26, 119.70, 119.17, 53.14, 29.13, 22.15, 13.86, 11.84; HRMS *m*/*z*: [M + Na]^+^ calcd for C_41_H_44_N_10_O_4_Na^+^, 763.3445; found, 763.3443.

## Supporting Information

File 1 Additional schemes and figures, general remarks, synthesis and characterization data, including copies of ^1^H and ^13^C NMR spectra.
