# Supplementary material for: A self-assembled photoresponsive gel consisting of chiral nanofibers
Source: Beilstein J Org Chem. 2018 Aug 1;14:1994–2001. doi: 10.3762/bjoc.14.174 (PMC6122117; doi:10.3762/bjoc.14.174)
Supplement: File 1 — Additional schemes and figures, general remarks, synthesis and characterization data, including copies of 1H and 13C NMR spectra. [file Beilstein_J_Org_Chem-14-1994-s001.pdf]

Supporting Information

for

A self-assembled photoresponsive gel consisting of chiral nanofibers

Lei Zou*, Dan Han, Zhiyi Yuan, Dongdong Chang and Xiang Ma*

Address: Key Laboratory for Advanced Materials and Institute of Fine Chemicals, School of Chemistry & Molecular Engineering, East China University of Science & Technology, Shanghai 200237, China

Email: Lei Zou- zoulei@ecust.edu.cn; Xiang Ma- maxiang@ecust.edu.cn

*Corresponding author

Additional schemes and figures, general remarks, synthesis and characterization data, including copies of ^{1}H and ^{13}C NMR spectra

Table of contents

1. General information S2
2. Synthesis and characterization data S3
3. Dates of ^{1}H, ^{13}C NMR and HRMS spectra S5
4. Fatigue resistance of compound **3** S8
5. FESEM image of compound **3** in benzene S9
6. References S9

General information

All reagents were purchased from commercial sources and used without further purification unless otherwise indicated. Compound **2** was prepared easily according to the literature method [1]. ^{1}H NMR and ^{13}C NMR spectra were recorded on a Bruker AM-400 spectrometer in DMSO-d_6 using tetramethylsilane (TMS) as the internal standard. High-resolution mass spectra (HRMS) were recorded on a Waters LCT Premier XE spectrometer using standard conditions. The UV–vis absorption spectra were obtained on a Varian Cary 100 spectrometer and a Varian Cary Eclipse (1 cm quartz cell was used). The UV irradiations (254 nm and 365 nm) were performed by a handheld UV lamp with an output power of 6 W. The light of 420 nm was performed by a lamp with an output power of 2 W/cm^2. SEM images were obtained by using Hitachi S-3400N scanning electron microscope and NOVA Nano SEM450 ultra-high-resolution field emission scanning electron microscope. The rheological experiment of the resulting organogel was carried out on MARS III instrument. CD spectra were recorded on a JASCO J-810 spectropolarimeter at 25 ºC.

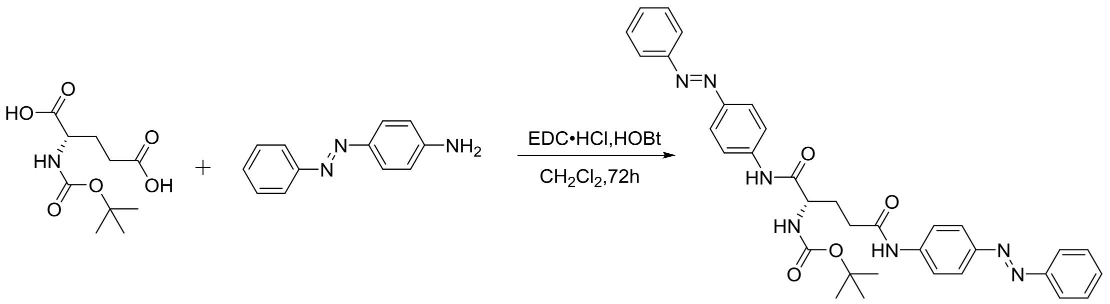

Scheme S1: Synthesis of compound 1.

1-Ethyl-3-(3-dimethyllaminopropyl)carbodiimide hydrochloride (EDC·HCl, 8.04 g, 0.044 mol) and 1-hydroxybenzotrizole (HOBt, 5.94 g,0.044 mol) were added into 200 mL CH_2Cl_2 solution of Boc-L-glutamic acid (5 g, 0.02 mol) and 4-aminoazobenzene (7.8838 g, 0.04 mol), then the obtained mixture was stirred at room temperature for 72 h under an Ar atmosphere. The obtained yellow solid was isolated by filtration and washed three times with CH_2Cl_2. The crude product was re-crystallized from THF/H_2O to yield compound **1** as a yellow solid (10.53 g, 87% yield). ^{1}H NMR (400 MHz, DMSO) δ 10.43 (s, 1H), 10.33 (s, 1H), 8.02 – 7.71 (m, 12H), 7.68 – 7.43 (m, 6H), 7.22 (d, J = 7.6 Hz, 1H), 4.18 (dd, J = 13.5, 7.9 Hz, 1H), 2.17 – 1.90 (m, 2H), 1.36 (d, J = 34.1 Hz, 9H). ^{13}C NMR (400 MHz, DMSO) δ171.44, 170.83, 155.43, 151.99, 147.58, 147.35, 142.35, 142.05, 131.04, 130.97, 129.38, 123.64, 122.32, 122.28, 119.50, 119.17, 78.23, 54.78, 32.92, 28.17, 27.07, 25.09. HRMS $[M + H]^+$ calculated for $C_{34}H_{36}N_7O_4^+$ 606.2829, found 606.2830.

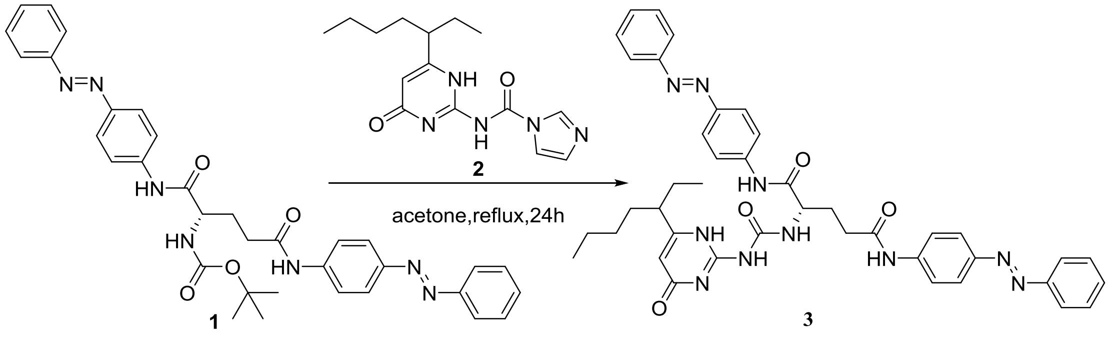

Scheme S2: Synthesis of compound 3.

Five mL trifluoroacetic acid was dropped into the 25 mL CH_2Cl_2 solution of compound **1** (1.0 g, 1.65 mmol), then the mixture was stirred at room temperature for 2 h under an Ar atmosphere. Evaporation of the resulting red solution was performed under reduced pressure, and a small amout of CH_2Cl_2 was added frequently into the bottle until the trifluoroacetic acid was removed entirely. The resulting yellow solid was added into an acetone solution of compound **2** (1.2205 g, 4.03 mmol) under Ar atmosphere, then the mixture was heated at reflux for 24 h. Evaporation of the resulting solution under reduced pressure and the further purification was carried out by column chromatography using CH_2Cl_2/CH_3OH (50:1, v/v) and CH_2Cl_2/CH_3OH (10:1, v/v) to afford yellow solid **3** (620 mg, 62.5% yield). ^{1}H NMR (400 MHz, DMSO) δ 11.29 (s, 1H), 10.67 (s, 1H), 10.34 (s, 1H), 9.68 (s, 1H), 8.30 (s, 1H), 8.01 – 7.68 (m, 12H), 7.68 – 7.43 (m, 6H), 5.77 (s, 1H), 4.64 (s, 1H), 2.22 (dd, J = 13.5, 6.0 Hz, 2H), 2.05 (dd, J = 13.6, 6.7 Hz, 1H), 1.55 (dd, J = 15.8, 10.0 Hz, 4H), 1.29 – 1.04 (m, 6H), 0.85 – 0.71 (m, 6H).^{13}C NMR (400 MHz, DMSO) δ170.51, 151.99, 147.79, 147.34, 142.30, 141.65, 131.11, 130.96, 129.41, 129.37, 123.57, 122.34, 122.26, 119.70, 119.17, 53.14, 29.13, 22.15, 13.86, 11.84. HRMS $[M + Na]^+$ calculated for $C_{41}H_{44}N_{10}O_4Na^+$ 763.3445, found 763.3443.

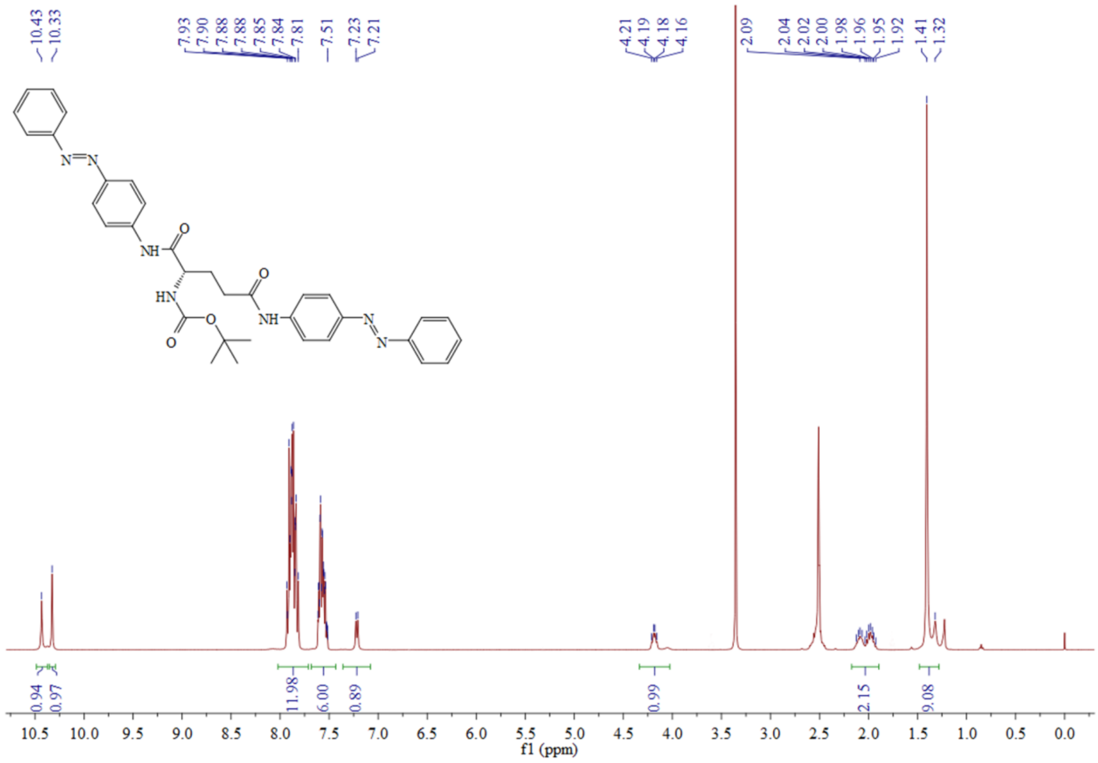

Figure S1: ^{1}H NMR of compound **1**.

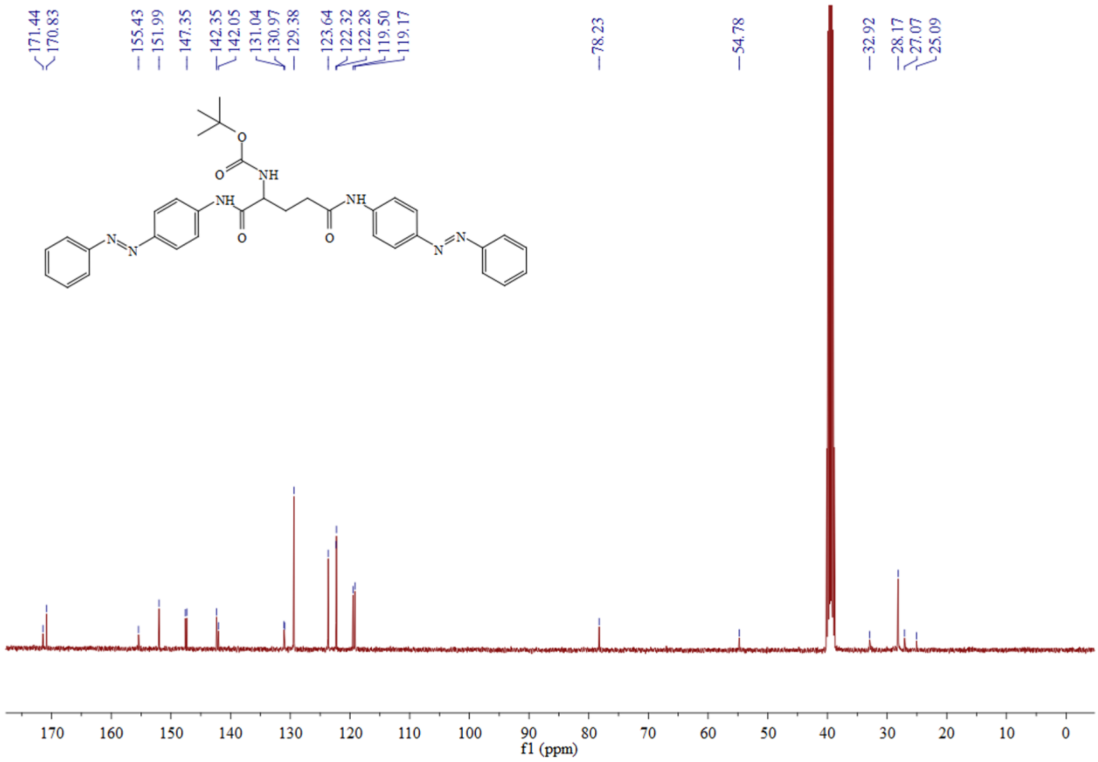

Figure S2: ^{13}C NMR of compound **1**.

Elemental Composition Report Page 1

Single Mass Analysis
Tolerance = 30.0 PPM / DBE: min = -1.5, max = 100.0
Element prediction: Off
Number of isotope peaks used for i-FIT = 2

Monoisotopic Mass, Even Electron Ions
167 formula(e) evaluated with 8 results within limits (up to 1 best isotopic matches for each mass)
Elements Used:
C: 0-53 H: 0-49 N: 0-7 O: 0-4

TIAN-H ECUST institute of Fine Chem 30-Nov-2017 16:02:43

TH-HD-005 17 (0.620) Cm (17:20) 1: TOF MS ES+ 1.35e+003

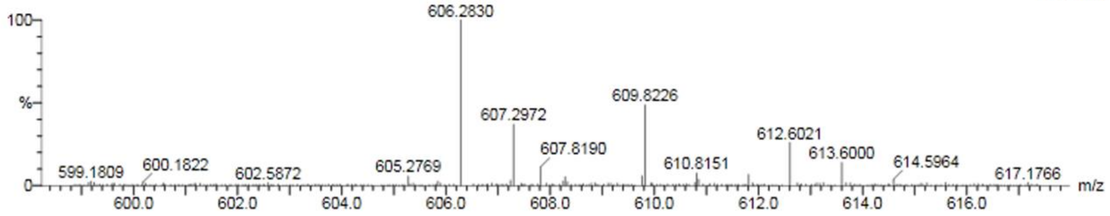

Minimum:				-1.5			
Maximum:		30.0	30.0	100.0			
Mass	Calc. Mass	mDa	PPM	DBE	i-FIT	i-FIT (Norm)	Formula
606.2830	606.2829	0.1	0.2	20.5	99.7	0.0	C34 H36 N7 O4

Figure S3: HRMS of compound **1**.

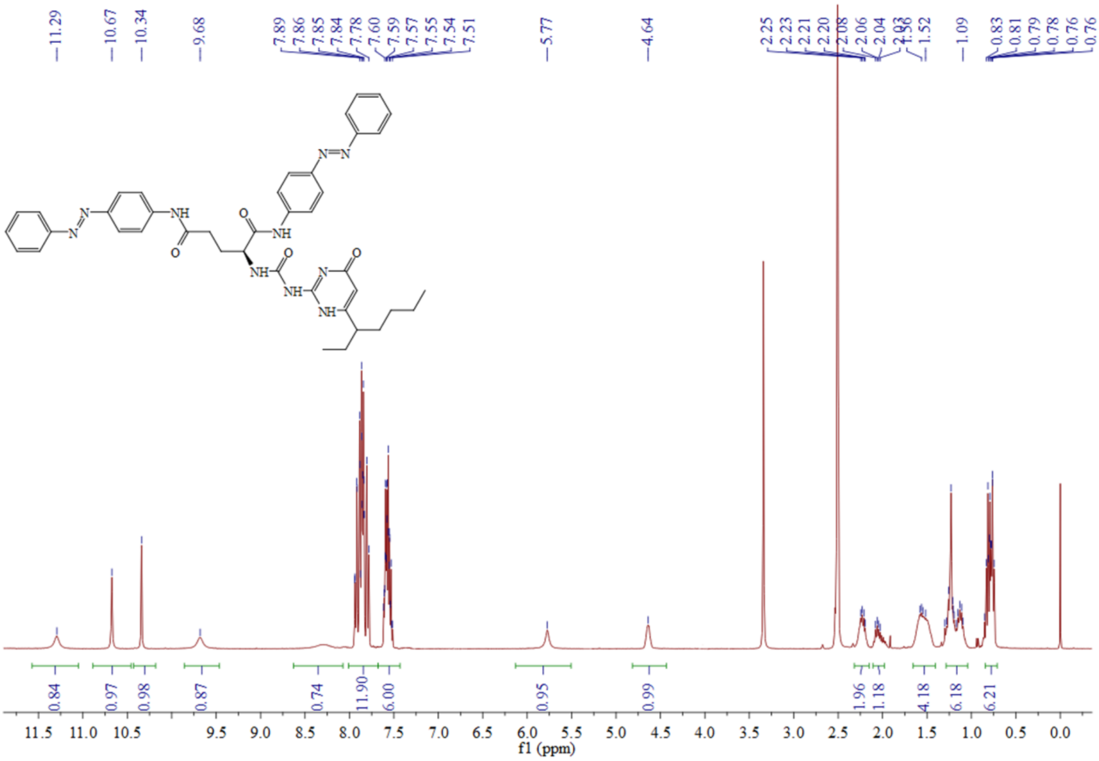

Figure S4: ^{1}H NMR of compound **3**.

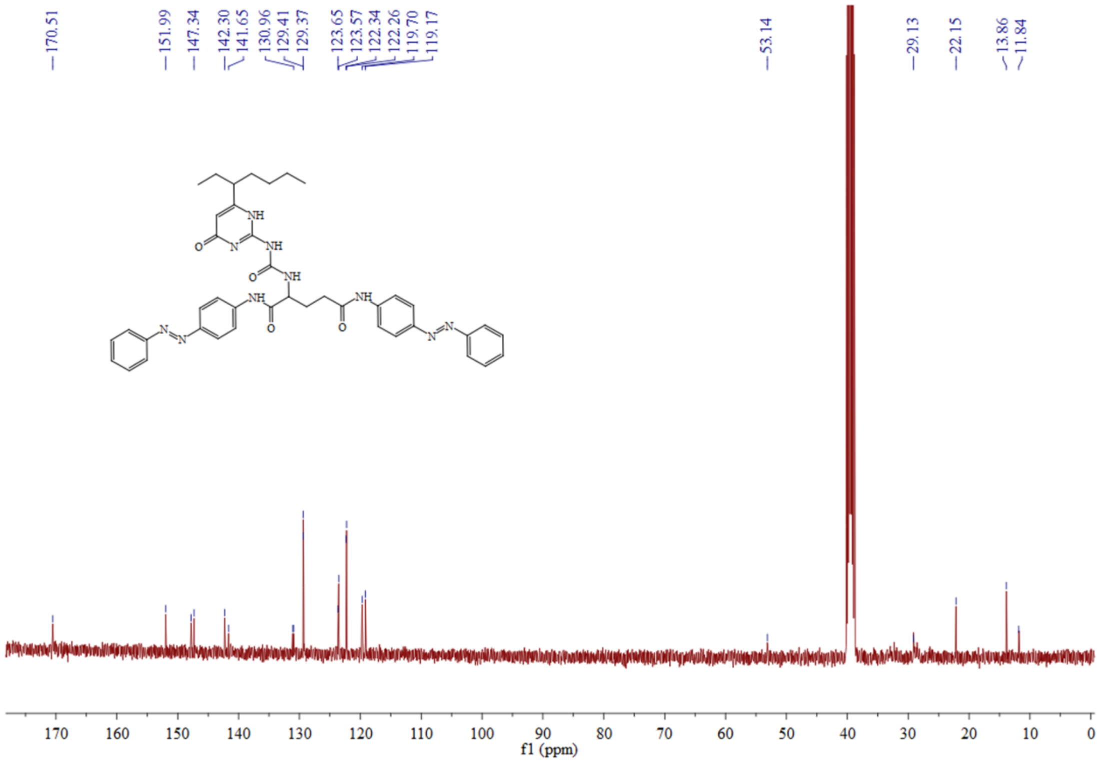

Figure S5: ^{13}C NMR of compound **3**.

Elemental Composition Report

Page 1

Single Mass Analysis

Tolerance = 5.0 PPM / DBE: min = -1.5, max = 50.0
Element prediction: Off
Number of isotope peaks used for i-FIT = 2

Monoisotopic Mass, Even Electron Ions
88 formula(e) evaluated with 1 results within limits (up to 50 closest results for each mass)
Elements Used:
C: 0-42 H: 0-46 N: 0-10 O: 0-4 Na: 0-1

ZOU-L
TH-HD-21 200 (5.055) Cm (186:202)

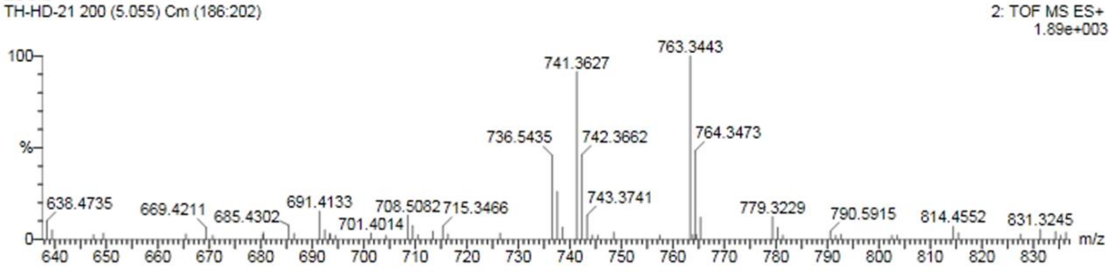

Minimum:				-1.5			
Maximum:		5.0	5.0	50.0			
Mass	Calc. Mass	mDa	PPM	DBE	i-FIT	i-FIT (Norm)	Formula
763.3443	763.3445	-0.2	-0.3	24.5	23.9	0.0	C41 H44 N10 O4 Na

Figure S6: HRMS of compound **3**.

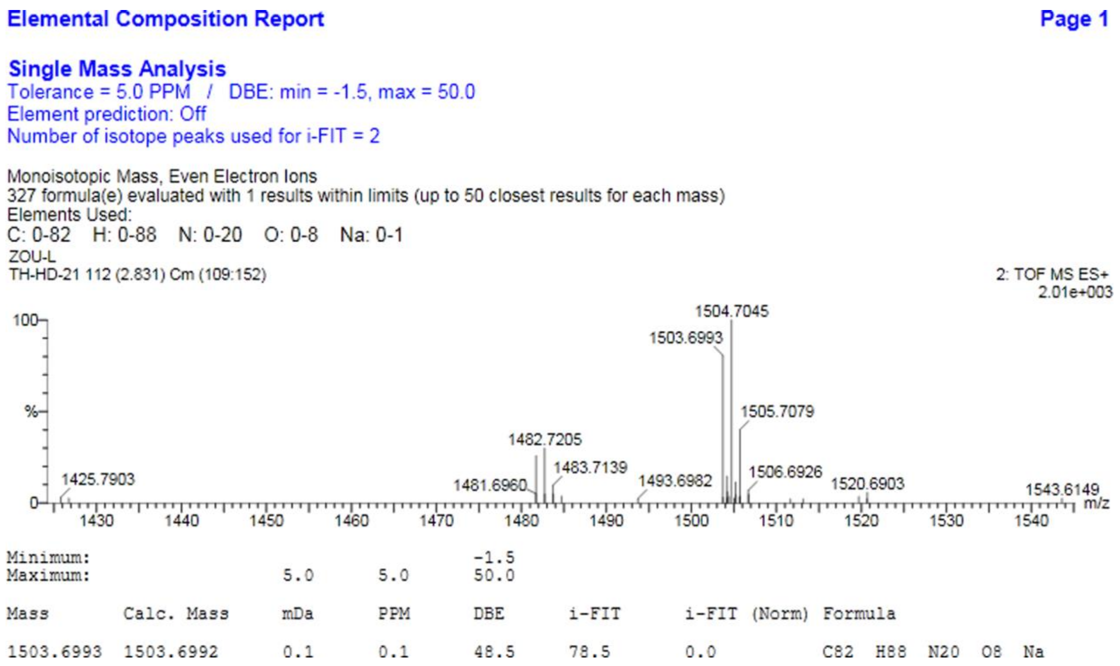

Figure S7: HRMS of compound **3**.

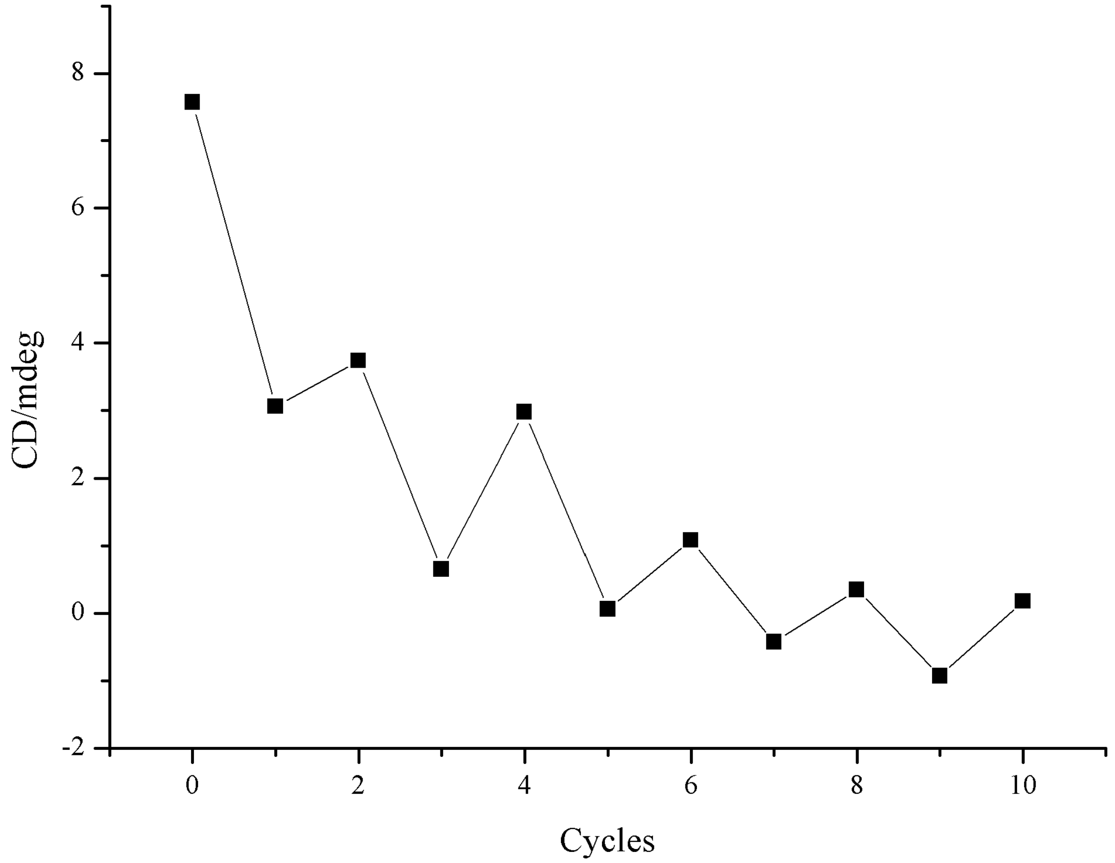

Figure S8: Fatigue resistance of compound **3** in tetrachloromethane(3 × 10^{-5}mol/L) under 365 nm/420 nm irradiation.

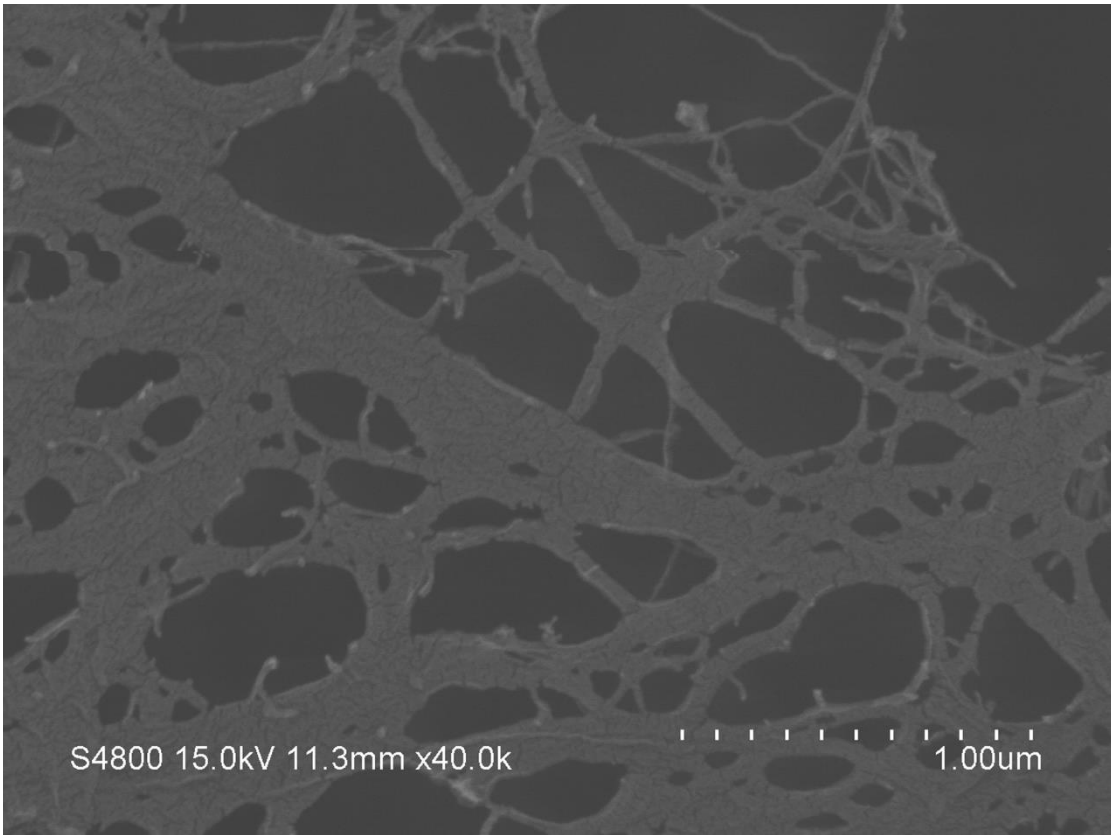

Figure S9: FESEM image of the microstructure obtain by assembled compound **3** in benzene.

References

1. Keizer H.; Sijbesma R.; Meijer EW. *European Journal of Organic Chemistry*, **2010**, 2553-2555. doi:10.1002/ejoc.200300752
